# Shaping Biological Knowledge: Applications in Proteomics

**DOI:** 10.1002/cfg.379

**Published:** 2004-03

**Authors:** F. Lisacek, C. Chichester, P. Gonnet, O. Jaillet, S. Kappus, F. Nikitin, P. Roland, G. Rossier, L. Truong, R. Appel

**Affiliations:** 1 R&D GeneBio 25 Avenue de Champel Geneva 1206 Switzerland; 2 Génome et Informatique Evry France; 3 Institute of Computational Science Swiss Federal Institute of Technology (ETH) Zurich Switzerland; 4 Swiss Institute of Bioinformatics and University of Geneva Geneva Switzerland

## Abstract

The central dogma of molecular biology has provided a meaningful principle for data integration in the field of genomics. In this context, integration reflects the known transitions from a chromosome to a protein sequence: transcription, intron splicing, exon assembly and translation. There is no such clear principle for integrating proteomics data, since the laws governing protein folding and interactivity are not quite understood. In our effort to bring together independent pieces of information relative to proteins in a biologically meaningful way, we assess the bias of bioinformatics resources and consequent approximations in the framework of small-scale studies. We analyse proteomics data while following both a data-driven (focus on proteins smaller than 10 kDa) and a hypothesis-driven (focus on whole bacterial proteomes) approach. These applications are potentially the source of specialized complements to classical biological ontologies.


**Comparative and Functional Genomics**
*Comp Funct Genom* 2004; **5**: 190–195.
Published online in Wiley InterScience (www.interscience.wiley.com). **DOI:** 10.1002/cfg.379

**Conference Review**

### Shaping biological knowledge: applications in proteomics

F. Lisacek[1,2]*, C. Chichester[1], P. Gonnet[3], O. Jaillet[1], S. Kappus[1], F. Nikitin[1], P. Roland[1], G. Rossier[1], L. Truong[1] and R. Appel[1,4]

[1]*R&D, Geneva Bioinformatics (GeneBio), Geneva, Switzerland*
[2]*Génome et Informatique, Evry, France*
[3]*Institute of Computational Science, Swiss Federal Institute of Technology (ETH), Zurich, Switzerland*
[4]*Swiss Institute of Bioinformatics and University of Geneva, Geneva, Switzerland*

*Correspondence to:
F. Lisacek, GeneBio, 25 Avenue de Champel, 1206 Geneva, Switzerland.
E-mail:
frederique.lisacek@genebio.com*

Received: 14 November 2003
Revised: 12 December 2003
Accepted: 18 December 2003

### Abstract

**The central dogma of molecular biology has provided a meaningful principle for data integration in the field of genomics. In this context, integration reflects the known transitions from a chromosome to a protein sequence: transcription, intron splicing, exon assembly and translation. There is no such clear principle for integrating proteomics data, since the laws governing protein folding and interactivity are not quite understood. In our effort to bring together independent pieces of information relative to proteins in a biologically meaningful way, we assess the bias of bioinformatics resources and consequent approximations in the framework of small-scale studies. We analyse proteomics data while following both a data-driven (focus on proteins smaller than 10 kDa) and a hypothesis-driven (focus on whole bacterial proteomes) approach. These applications are potentially the source of specialized complements to classical biological ontologies. Copyright © 2004 John Wiley & Sons, Ltd.**

**Keywords:** **bioinformatics; proteomics; data integration; annotation; ontology**

### Introduction

In many bioinformatics applications sequences constitute the core data and, consequently, sequence annotation is a crucial task. Various more or less independent sources are available for the manual or automated extraction of relevant information related to a sequence or a collection thereof. In the specific context of proteomics, the focus is on proteins. Reliable annotation involves cross-checking extracted pieces of information, possibly complemented with mass data, while identifying contextual constraints that characterize protein structure, function and modifications. Well-annotated proteins are clearly valuable for interpreting experimental results.

Automated procedures for gathering multisource information are commonly acknowledged as data integration schemes. More precisely, integration entails producing a synthetic picture, which involves two distinct tasks: centralizing information in one given location and blending this information in, given an underlying principle.

A mountain of technical difficulties relative to solving format compatibility problems, implementing cross-talk between diversely configured web servers, etc., has channelled most bioinformatics efforts towards tackling the first issue and, often enough, information is only piled up in one location. In fact, accumulating properties of protein sequences is as informative as listing ingredients for cooking. The recipe is missing; that is, quantification and chronology. Furthermore, the cooking analogy also emphasizes the necessary *blending-in* step of integration, which is too often overlooked. In fact, the analysis of texture is the hidden key to blending mixtures in. In very much the same way that some ingredients mix or do not mix smoothly, multisource

Copyright © 2004 John Wiley & Sons, Ltd.

data complement or do not complement each other. The texture, or the *granularity*, of information sets the way information should be merged to become synthetic. Defining and understanding varying grain sizes of information allows the definition of levels and then, the use of zooming operations that are essential for exploratory purposes.

Operations that define weighing, ordering and merging of information require regular consistency checks. Consistency should not only correspond to logical soundness but also to biological knowledge, e.g. the known transitions from a high-level chromosome to a low-level protein sequence set an appropriate scheme for structuring gathered pieces relative to genomic sequences. DNA sequences are consistently mapped with multiple transcripts and related to spliced introns and translatable exons. Gene loci along a chromosome are set as references for defining zooming operations (i.e. points where information is blended in). In this case, integration reflects meaningful principles of molecular biology and an identified chronology of events. The integration of genome data was quite efficiently implemented, e.g. in EnsEMBL [4]. Unfortunately, our lack of understanding of the laws governing protein folding and interactivity hinders the design of appropriate and comprehensible templates for integrating proteomics data. Moreover, the various levels that allow zooming in and out cannot be straightforwardly defined.

So far, the Gene Ontology [1] remains the most popular initiative for structuring interpretable protein data. Knowledge is currently unevenly represented due to the novelty of this concerted effort and the limitations of understanding in biology.

We have undertaken several studies to address mainly the second aspect of proteomics data integration. The common purpose of these studies is to determine relevant intermediary concepts, i.e. possible integration levels [10]. This entails testing various representations of the same entities in set contexts. Various representations require consistency checks for navigating between levels. These topics are summarized in what follows.

### Biases and approximations

### Heritage

We assume our starting point is a self-contained proteome, i.e. the translation of a complete genome or a comprehensive set of proteins extracted from a specific tissue. A number of preliminary questions need to be addressed prior to setting meaningful principles of integration. A first step is a fact-based assessment of what is known about the particular set of proteins and the bioinformatics resources (databases and tools) attached to the generation of these facts. Indeed, uneven data production spread over many years implies that a lot can be known about some proteins and hardly anything about others. For instance, protein structure databases contain more proteins that form crystals easily than transmembrane proteins, which are a crystallographer's nightmare.

In fact, for many years, data resources have promoted rapidly increasing numbers of entries reflecting an incessant production of data. Moreover, efforts were often guided by criteria such as maximized coverage of topics, species, etc., i.e. tending towards exhaustiveness. As a result, in a lot of large databases, information is often redundant; alternatively, it is averaged and specificity is lost. Exhaustiveness is a criterion that long meant *ever-increasing size* and was applied to counting objects. The recent release of genome data has modified this view. An exhaustive set is now accepted as including a small number of entities. The number of entries of a genome or proteome database is not expected to grow but information related to each entry is supposed to be dug into. Now, properties of objects are actually expected to be exhaustive. Database expansion has shifted from breadth to depth and from objects to properties for a clearer picture of biology.

### Data

As a consequence of the former definition of exhaustiveness, biological data were often collected because they were available as opposed to being deliberately selected. Resulting trends can be identified in databases, e.g. most protein family databases are biased towards enzyme-related domains, given the traditional tight knit between structural domains and enzymatic activity in protein studies.

Once a bias is made explicit, its impact on the quality of annotation can be assessed and relevant questions can be set. As a corollary, available information is weighed to counteract the effect of that bias. Established weaknesses at

Copyright © 2004 John Wiley & Sons, Ltd.

the level of data led us to focus on proteins of unknown function in complete bacterial proteomes. While assuming that proteins are modular, we have followed a *hypothesis-driven* approach to corroborate scattered information.

### Models

Heterogeneous data has also affected prediction methods that were defined to specify protein features (structure, subcellular localization, interacting partners, posttranslational modifications, etc.). A predictive model reflects a current state of knowledge, and incomplete knowledge inevitably gives rise to a certain proportion of ill-defined concepts (quite naturally, though, new knowledge helps to correct or refine such concepts). Such a situation enforces approximations, which in turn conditions the performance of bioinformatics software.

Identified weaknesses at the processing level led us to select a topic in need of dedicated processing. Such is the case of proteins of small size (<10 kDa), which cannot be detected by any of the programmes used for predicting coding regions in genomic DNA [11]. Aspects of RNA splicing mechanisms that remain unclear hamper the definition of a reliable model. As a result, common prediction schemes impose restrictions on protein size, and short proteins are mostly identified by experimental means. We have focused on the annotation of families of small, secreted proteins. This approach can be considered as *data-driven*.

### Texts

It is commonplace to state that biological knowledge is textual knowledge. Annotations are written as texts. The best justification of an annotation is a published article. Halfway between the data-driven and hypothesis-driven approaches, we rely on text analysis to supplement our investigations.

### Data-driven approach: study of small proteins

Fast processing is particularly necessary, given the current intensive effort for sequencing complete genomes. However, speed and quantity are often achieved to the detriment of quality. This issue is well introduced in Gattiker *et al.* [6], where high standards for producing reliable protein annotations are set. Among others, a relevant strategy involves gathering sequences into consistent families while carefully defining similarity criteria. Grouping criteria do not necessarily reflect a global similarity of amino acid sequences. Some proteins can be functionally equivalent although structurally very diverse (including at the sequence level).

The creation of a new generation of curated and comprehensive data resources has emerged as a possible answer to the critical situation of information overflow. Those resources include non-redundant and exhaustive data as well as appropriate analysis tools to explore, visualize and analyse the many aspects of data.

Our contribution (manuscript in preparation) highlights some of the capabilities to identify and cross-link context-sensitive information. In particular, the definition of viewpoints and the possibility of corroborating these viewpoints. We set the reconciliation of various viewpoints via consistency checks as a means of characterizing a context.

### Hypothesis-driven approach: protein modularity

As yet another consequence of data collection efforts spread over time, current protein families are a mosaic of functional, structural and sequence features, as visible in the federated InterPro web resource [12]. Protein modularity is extensively highlighted in this resource, where instances of proteins belonging to several families are displayed. Similar emphasis is put on modularity in a more recent contribution named CDART [7]. However, in both instances, most statistics on protein properties are performed using large datasets of proteins from all possible origins, which makes the appreciation of the potential subtlety of protein characteristics difficult. Large variations in family size and species coverage generate very uneven entries in protein family databases, where information piles up with no preset priority.

Simple and elementary questions for which the answers could not be found in the literature were tentatively addressed in Nikitin and Lisacek [13]: how is protein family membership distributed across a single proteome? How combinatorial is such a distribution? How variable is a proteome content in that respect? Moreover,

Copyright © 2004 John Wiley & Sons, Ltd. 

considering that a substantial part of the information detailed in protein families is enzyme-related for known metabolic pathways, less documented families involving membrane-related activities or unqualified function were focused upon.

Examining each proteome independently and comparing the occurrences of modular combinations led us to: (a) identify discriminating properties between bacteria that could be generalized; (b) study various modular combinations and formulate hypothesis on functional implications; and (c) set the basis of a similarity measure between proteomes.

### A mixture of both approaches: hidden polymorphism in literature databases

In many instances mass data confirm the presence of various forms of a protein. In fact, depending on the organism and the tissue under investigation, from two to over 10 protein forms can be identified in a given sample (see the index to 2-D PAGE databases at **www.expasy.org/ch2d/2d-index.html**). The term 'alternative form' is used in the following text to describe any product of a gene, including its close duplicated copies. Given a subset of proteins of interest, possible isoforms can be itemized and related to each other, as quoted in the literature. Moreover, the relationship between a given isoform, e.g. a splice variant, and the conditions of its production (pathological/non-pathological, early/late development, tissue specificity, etc.) can be of value in rationalizing the presence or absence of this specific gene product. Whenever sequence variations can be matched with experimental mass data, mining the literature contributes to designing and building up a documented source describing possible scenarios of alternative protein form generation. Observed co-occurrence of two or more protein forms in such a source would allow the inference of complementary knowledge. Indeed, the presence of an alternative form, e.g. known to be specific to an ageing cell, along with that of an alternative where exceptions have been noted, could lead to relating the exceptions to a particular developmental stage and spur further tests.

In Chichester *et al.* [3] directions and guidelines were sketched for tracking the origin of alternative forms as identified in translated EST sequences, in an attempt to tag the specificities of sequence data in the literature. Simple consistency checks in databases helped to demonstrate the existence of diverse alternative forms, which can then be matched in publications. Our point was that the identification of protein–protein interactions should in reality be that of identifying (alternative) form–form interactions when rationalizing protein function.

The overall goal of proteomics studies is to generate a detailed description of the molecular players within a process. The identification of protein alternative forms provides a basis for further analysis of molecular events in the correct context.

### Basis for a principle

The basis of integration for protein data should obviously express transitions from sequence to structure. These transitions are likely to involve diverse properties of proteins and hence various representations. A number of more or less flexible description schemes have been set to represent and extract various properties of protein sequences. Proteins have been considered as successions of amino acid properties (e.g. [16]) or characterized by regular expressions (e.g. [2]), weight matrices (e.g. [15]) or profiles (e.g. [5,6]), etc. Numerous *ad hoc* or formal methods yielding calculations of characteristic indices, weights and scoring functions have been published. It is now textbook material. These different frameworks all converge towards the existence of consistent internal units such as domains or, more broadly, signatures. Such an assumption is substantiated by the idea that evolutionary units might be shorter than a complete gene [14]. We have further explored the possible mapping between different representations of proteins through the study of protein motifs [8] and protein modules [13].

We are currently in the midst of elucidating possible rules justifying the transition between combinations of amino acids to combinations of groups of amino acids (modules). We are testing the relevance of this hypothesis in the context of proteome comparison, as illustrated in Figure 1.

As mentioned in the introduction, different grain sizes of information allow zooming operations. We study these levels from the sequence to the structure level with bacterial proteins and from

Copyright © 2004 John Wiley & Sons, Ltd. 

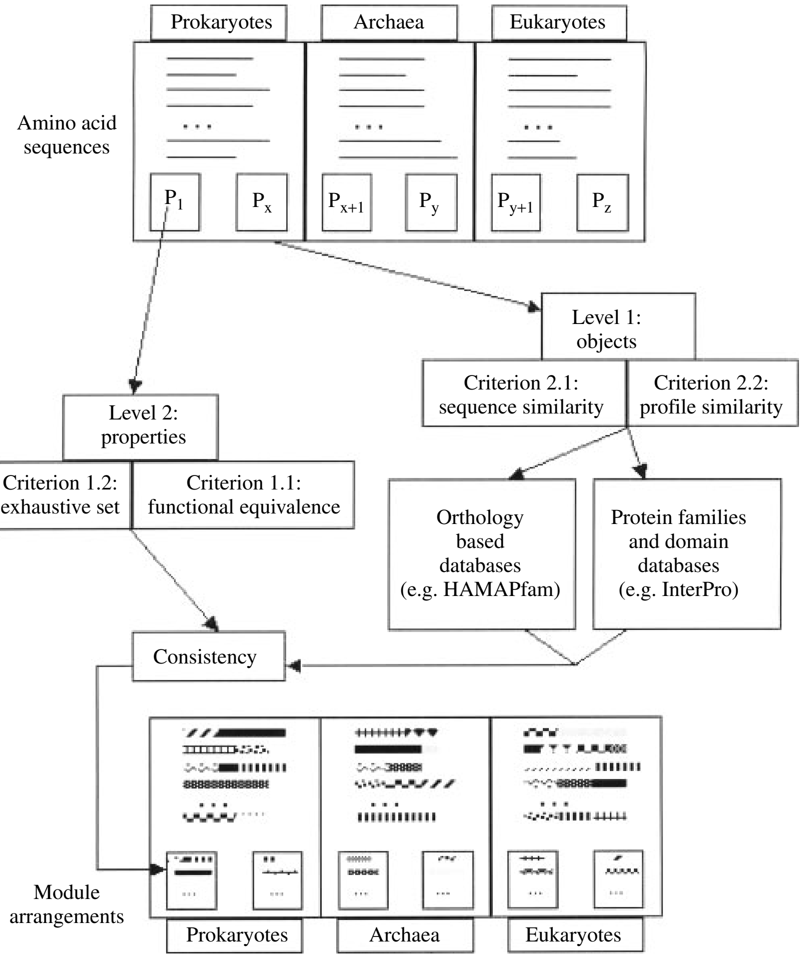

**Figure 1.** An 'idealized' mapping between amino acid sequence databases and modular combinations within proteins. Databases are partitioned with respect to major species reigns; smaller rectangles symbolize complete proteomes (denoted $P_1, P_x, \ldots, P_z$). Consistency checks involve several levels and criteria. At the level of objects, i.e. sequences, consistency depends on diverse similarity measures. At the level of properties of objects, consistency is related to biology. A proteome is supposed to be consistent, as it is an exhaustive set. A set of functionally equivalent proteins is consistent from the biochemical function standpoint. This heterogeneous information is corroborated via different data resources. This situation describes the option of using a module-based similarity measure for comparing proteomes [13]

the protein to the process level in textual analysis. These applications are potentially the source of specialized complements to classical biological ontologies.

### Concluding remarks

The work described and discussed above attempts to show that the most frequent and abundant information tends to hide the most relevant information. The currently available databases are typically resourceful but too rich. Shaping biological knowledge in proteomics definitely involves selecting and cross-linking information, but also identifying key levels of information for understanding the rules of precedence of the various pieces of information.

### Acknowledgements

We wish to thank the entire GeneBio team within which we work in ideal conditions as well as the Geneva groups of the Swiss Institute of Bioinformatics for the regular and productive interactions we benefit from.

Copyright © 2004 John Wiley & Sons, Ltd. 

### References

1. Ashburner M, Ball CA, Blake JA, *et al*. 2000. Gene ontology: tool for the unification of biology. The Gene Ontology Consortium. *Nature Genet* **25**(1): 25–29.
2. Bairoch A. 1991. PROSITE: a dictionary of site and patterns in proteins. *Nucleic Acids Res* **19**: 2241–2245.
3. Chichester C, Nikitin F, Ravarini J-C, Lisacek F. 2003. Consistency checks for characterizing protein forms. *Comput Biol Chem* **27**(1): 29–35.
4. Clamp M, Andrews D, Barker D, *et al*. 2003. Ensembl 2002: accommodating comparative genomics. *Nucleic Acids Res* **31**(1): 38–42.
5. Eddy SR. 1998. Profile hidden Markov models. *Bioinformatics* **14**(9): 755–763.
6. Gattiker A, Michoud K, Rivoire C, *et al*. 2003. Automatic annotation of microbial proteomes in Swiss-Prot. *Comput Biol Chem* **27**: 49–58.
7. Geer LY, Domrachev M, Lipman DJ, Bryant SH. 2002. CDART: protein homology by domain architecture. *Genome Res* **12**(10): 1619–1623.
8. Gonnet P, Lisacek F. 2002. Probabilistic alignment of motifs with sequences. *Bioinformatics* **18**: 1091–1101.
9. Gribskov M, McLachlan AD, Eisenberg D. 1987. Profile analysis: detection of distantly related protein. *Proc Natl Acad Sci USA* **84**: 4355–4358.
10. Lisacek F. 2003. Shaping biological knowledge. *Pharmacogenomics* **4**(1): 5–8.
11. Mathe C, Sagot MF, Schiex T, Rouze P. 2002. Current methods of gene prediction, their strengths and weaknesses. *Nucleic Acids Res* **30**(19): 4103–4117.
12. Mulder NJ, Apweiler R, Attwood TK, *et al*. 2003. The InterPro Database, 2003 brings increased coverage and new features. *Nucleic Acids Res* **31**: 315–318.
13. Nikitin F, Lisacek F. 2003. Investigating protein domain combinations in complete proteomes. *Comput Biol Chem* **27**(4): 483–497.
14. Ponting CP, Russell RR. 2002. The natural history of protein domains. *Annu Rev Biophys Biomol Struct* **31**: 45–71.
15. Staden R. 1988. Methods to define and locate patterns of motifs in sequences. *Comput Appl Biosci* **4**: 53–60.
16. Taylor WR. 1986. The classification of amino acid conservation. *J Theor Biol* **119**: 205–218.

Copyright © 2004 John Wiley & Sons, Ltd.
